# Designing Iranian hospital organizational charts: Global comparisons

**DOI:** 10.1371/journal.pone.0300985

**Published:** 2024-03-27

**Authors:** Mostafa Amini-Rarani, Somayeh Mokhtari, Mohammad Akbari, Zahra Zamani, Somayeh Mahdiyan

**Affiliations:** 1 Social Determinants of Health Research Center, Isfahan University of Medical Sciences, Isfahan, Iran; 2 Health Management and Economics Research Center, Isfahan University of Medical Sciences, Isfahan, Iran; Alexandria University Faculty of Nursing, EGYPT

## Abstract

**Background:**

Hospitals should have effective and efficient organizational charts to face the changing healthcare environment. Thus, for this purpose, the present study seeks to compile an organizational chart for Iranian hospitals.

**Materials and methods:**

The present study was conducted in two phase overview and qualitative (using focus group discussion). In the overview phase, the organizational charts of hospitals were analyzed in terms of complexity (i.e., degree of horizontal and vertical separations), and the initial hospital organizational chart was developed based on the results. Subsequently, experts were interviewed in a focus group discussion to finalize and validate the initial organizational chart.

**Results:**

The final organizational chart was designed to contain features such as internal divisions, specialization, reduction of organizational hierarchies, expansion of supervision scope, and moderate-sized organizational pyramid.

**Conclusion:**

Using designed organizational chart would eliminate the redundant managerial levels since it reduces organizational hierarchies to two levels of management, expands the supervision scopes, fosters a moderate-sized organizational pyramid, and catalyzes communications.

## Background

The recent management transformations emerging in the current dynamic age are steering managers to adopt flexible, new strategies and new management attitudes to overcome the challenges in the exterior environment [1].

In this regard, designing an appropriate organizational structure is essential after determining organizational goals and strategies [2]. Designing an appropriate organizational chart can help acquire advantages stemming from agility through focusing on the employees, innovation, and the creativity resulting from their effective collaboration, and would empower organizations to adopt features such as flexibility, quick reactions in the face of environmental change, and responsiveness to predictable and unpredictable changes [3]. Organizations that prepare themselves to make the necessary changes and take advantage of development models and techniques in organization structure will achieve competitive advantages over other organizations. Thus, comprehension of organizational structure is the best way to face the complexities and changes in the function of organizations [1].

Designing a suitable organizational structure plays a major part in the efficiency and performance improvement of human resources in any organization and will lead to the effective implementation of strategies, the accomplishment of organizational goals, the identification of roles and tasks of various departments, facilitation of communication and intra-organizational connections, and increased efficiency in providing services [4]. Moreover, understanding and designing a proper organizational chart can help achieve goals such as economic and effective performance of the organization, adjustment to the effects of environmental changes, and social satisfaction for the staff. It can thus be suggested that the organizational structure is the most important parameter affecting efficiency in the organization and influencing other parameters as well [5].

Since achieving organizational goals is the most important success factor for any organization, the manager must design a structure that fits the organizational goals and strategies [2] and facilitates the implementation of strategic plans [6]. Designing the organizational structure can be defined as a decision-making process steering the organization toward its vision, values, mission, and goals [6]. Of course, the organizational structure model is not permanent and may change depending on environmental contingencies and organizational tasks [7].

The organizational structure comprises two content and structural dimensions. The content dimensions include areas such as strategy, culture, technology, and environment, which represent the organization’s position and influence the structural dimensions [2]. The structural dimensions, on the other hand, represent the innate features of an organization and provide the basis based on which organizations can be measured and compared [8]. Various classifications have been proposed for the structural dimensions, but the most agreed-upon includes categories of complexity, formality, and centrality [2]. The intensity or weakness of each of these three structural dimensions is crucial and affects the organization’s overall structure and decision-making [4].

Formality marks the extent of bureaucracy, regulation, rules, and communications in the organization, while centrality is concerned with the delegation of authority in the organization and determines who has the right to make decisions. Finally, complexity is the degree of individuals’ task specialization and is defined and measured by the number of locations in which the work is being conducted, the number of positions, and the number of hierarchies performing various tasks [9].

According to Robbins’ theory, three variables of "formalization", "complexity" and "concentration" of organizational structure can be analyzed. Formalization indicates the importance of the level of familiarity of the employees with the values and missions of the organization and the level of attention given by the organization to its employees. Centralization in the organization determines the extent to which employees and operational managers are authorized to make the decisions. Complexity illustrates the horizontal, vertical, and geographical separation in an organization. The degree of separation can affect the level of communication the employees have along with the employee’s perception of work [10].

In this study, we used the criteria of vertical and horizontal separation in the dimension of complexity. Horizontal separation indicates the degree of separation between organizational units based on the position of the organization’s members using criteria such as the number of units, different job titles, scope of professional activity, and repetition of duties. Vertical separation refers to the height or depth of the organizational hierarchy, and its criterion is the height of the organizational structure or, in other words, the number of management levels [11].

It must be mentioned that a distinction is to be made between organizational structure and organizational chart. Organizational structure is a more general term represented by the organizational chart. The organizational chart is a visible representation of the formal communication channels and working groups [12]. One could suggest that the organizational chart is a graphical demonstration of the organizational structure. In large organizations consisting of various units and departments, such as hospitals, organizational charts can be used to specify how the components of the organization work together and how the employees coordinate their performance to achieve organizational goals [13].

One of the challenges that managers, experts, and policymakers are currently facing in terms of hospital administration is the identification of ways to improve care through the improvement of the organizational chart and communications in the hospitals [14] since hospitals are dynamic organizations operating in a constantly changing environment [15]. Moreover, since the organizational structure is one of the crucial factors affecting employee empowerment, the formal relationships between the individuals, job positions, job descriptions, resource allocation, rules and regulations and their implementation, and coordination between various activities and units will not form if hospitals fail to develop a standard and appropriate structure [16]. A non-standard organizational structure can cause excessive formality and complexity, centralism, planned behavior in the form of cumbersome regulations, excessive division of work, negligence of employee opinions, poor responsibility fulfillment, inflexibility, excessive rules and regulations, complex network of relationships, and ultimately, restrictive conditions for employee empowerment [17].

Each organization, whether small or large, has an organizational chart that is expected to facilitate efficient management of the wards [18]. However, there have always been problems in the formation and stability of a dynamic organizational chart model in Iranian hospitals, to the point that no appropriate organizational chart has been designed so far. Moreover, various names have been considered for each hospital, given the nature of their duties and based on mere taste. Although "Collection of Organizational Engineering Regulations and Engineering and Job Evaluation" book, issued by the Iranian Ministry of Health and Medical Education, has defined and classified various types of hospitals [19], no procedural unity is observed in the naming and organizational chart of hospitals given the nature of their tasks, activities, and goals. Thus, the present study seeks to design an appropriate organizational chart for various hospital types in terms of their tasks (teaching and non-teaching hospitals) and type of activity (general or specialized). Results of the present study can help develop a similar organizational chart and lead to procedural unity in hospital naming given the type of tasks and activities, thus facilitating the comprehension of the chain of command, speeding up organizational communication, and removing redundant bureaucracies.

## Materials and methods

The current study was conducted in two phase’s overview and qualitative. In the overview phase, the organizational chart of different hospitals was first overviewed to design an initial organizational chart model for Iranian hospitals. In the second phase, focus group discussions (FGD) were conducted to finalize the initial model.

### Setting

According to the "Collection of Organizational Engineering Regulations and Engineering and Job Evaluation" published by the Iranian Ministry of Health and Medical Education, a hospital is a medical institute established to treat inpatients and outpatients using therapeutic and diagnostic facilities around-the-clock and has different types [19]:

1. *General Hospital*: This is a second-grade or third-grade healthcare institute in the healthcare system with at least four departments of internal, obstetrics, pediatrics, and general surgery, as well as a pharmacy, laboratory, radiology, emergency, and nutrition units, all of which are integral to the hospital. Hospitals will be authorized to operate based on the establishment and technical permits issued separately by the Deputy Minister of Treatment.
2. *Specialized hospital*: Operates in one or several specialized medical fields.
3. *Teaching healthcare center*: A hospital providing clinical education to medical sciences students in addition to diagnostic and therapeutic services to patients under a permit from the Deputy Minister of Education. Most specialist physicians at these hospitals are faculty members at the schools of medicine.

This classification based on tasks (teaching and non-teaching) and activities (general and specialized) has been determined by the Ministry of Health and Medical Education. The present study has investigated hospitals’ organizational charts based on the classification mentioned above in Iran, Also, to know more about the organizational charts of different countries and to examine their models and model their strengths for Iran, we selected a few countries including the USA, Canada, Egypt, Philippines according to the entry criteria.

### Study design

This study was conducted in the two following phases (**1. Overview** and **2. Qualitative**):

**Phase one: Overview** of hospital organizational charts

We reviewed different countries with different levels of development and income as in Iran there are provinces with different levels of development and income. So, through reviewing countries with different contexts we reached a comprehensive and deep understanding of hospital organizational charts in various contexts.

*Step 1*: *Data collection*. At this step, hospitals’ organizational charts were reviewed based on the following criteria:

1. Access to the organizational chart is available on the hospital website
2. The existence of an up-to-date organizational chart on the hospital website for the period from 2014 to 2021
3. The availability of notable points to learn from based on the initial search and researchers’ opinions, such as organic, flexible, and flat charts, specialization, shorter hierarchies, and division of the specialized units [20].

Accordingly, based on these criteria, the followings stages were carried out:

1. Searching hospital websites
2. Examination of the organizational chart available on hospital websites
3. Checking whether the organizational chart is up-to-date
4. Examining the organizational chart based on the specifications of effective and efficient charts

Thus, in t phase, the organizational charts of teaching and non-teaching, general, and specialized hospitals in various countries were reviewed in terms of complexity (vertical and horizontal separation).

*Step 2*: *Data analysis*. According to the results obtained from the previous step, all the selected organizational charts were analyzed, and the initial hospital organization chart was designed based on complexity and divided by the tasks and activities of the hospital [19].

#### Phase two: Qualitative

For this phase, Focus Group Discussion (FGD) was used to finalize and validate the initial hospital organizational chart. The following steps were taken to carry out FGD [21]:

*1-FGD design*. At this stage, the main goal behind the design of an organizational chart was specified. These goals include improving the efficiency and performance of human resources, effectively implementing strategies to accomplish organizational objectives, specifying the duties and responsibilities of different departments, promoting communication and collaboration, and enhancing service delivery within the organization. Then a list of the features of appropriate and efficient organizational charts was prepared as a guide for each session of the FGD. These features include specialized units, shorter hierarchies, and flexible organic and flat charts.

Then, 8 experts were selected from the organizational chart design specialists and heads of organizational engineering departments in Iranian universities in 2021 through purposive sampling seeking to improve group dynamicity and the synergy between the participants in data production [21]. The inclusion criteria for experts were their academic degree (bachelor’s and higher) and adequate experience (five years or more in the field of hospital organizational cart design). All of the selected members participated in the interview.

Then, the results of the first phase (overview) and the basis for the design of hospital organizational charts were explained to the experts through Google Meet and WhatsApp applications to implement the FGD, and each was asked to express their individual opinions [21]. We had a series of meetings until the participants came up with a fresh proposal for the hospitals’ organizational chart, and we reached the point of data saturation.

Credentials and occupation of the research team at the time of the study was as follow. M.A.R hold Ph.D. in health policy and worked as assistant professor. S.M. has nursing credentials (MD), worked in various hospitals and experienced as an educational supervisor in hospital. M.A. hold nursing credentials (MD) and was the head of the organization development management. He also had work experience in various hospitals. Z.Z. hold nursing credentials (MD) and has worked in several hospitals. She also served as the head of the business engineering unit. S.M. (corresponding author) educating as Ph.D. candidate in healthcare management and worked in organization development management.

*2-Data collection*. Experts’ opinions regarding the organizational position of each unit based on the vertical and horizontal separation criteria were shared over 12 two-hour sessions. Notes were taken during the group discussion sessions, and the sessions were also voice-recorded after receiving the participants’ consent [21]. In the group discussion sessions, S.M. was in charge of note-taking while Z.Z. handled audio recording and session implementation.
*3-Analysis and conclusion*. At this stage, the initial chart designed in the previous stage was demonstrated and explained to focus group discussion members to vote and rank the opinions regarding organizational positions in each unit based on its tasks considering vertical and horizontal separation criteria. The members discussed the initial chart over three two-hour sessions. Each of the members first gave their opinion regarding the position of managers and units working under each management. After the discussion and settling of disagreements (by voting), the agreed-upon opinions of the group were written on the board, based on which the necessary changes were made to each management position and the units working under it in the initial chart. Thus, the final chart was compiled. It must be noted that consensus was reached on opposing opinions through voting, and dominant opinions were determined based on voting results. M.A.R. identified the opinions of the majority of members based on the voting results and compiled the final chart.
*4-Report compilation*. Results of the FGD sessions were integrated and compiled in a report. This report included key quotes from the experts as well as their information, including their academic degrees, organizational experience, and background. All the opinions were shared in a process called member review to validate the results, which increased the validity of the designed chart [21]. We followed the EQUATOR guidelines for reporting data, specifically the Quality Research Checklist (COREQ), to ensure clear and comprehensive reporting of FGD. To achieve this, we used all 32 criteria listed in the COREQ checklist [22].

The steps of the study are shown in S1 Fig.

### Ethics approval and consent to participate

This study received the required ethics approval from the Isfahan University of Medical Sciences Research Ethics Committee, Isfahan, Iran, with ethical code no. IR.MUI.NUREMA.REC.1400.028.

All study participants provided verbal informed consent to participate. During FGDs, verbal consent was obtained from the participants before the recording of the FGD meetings. Participants were told that their participation was voluntary, and they were assured that their comments would be confidential. All methods were carried out with relevant guidelines and regulations

## Results

The results are presented in the following two sections.

### 1-Overview of organizational chart of hospitals

The following results were obtained based on the overview in this phase:

1. Accessing the websites of 206 hospitals
2. Reviewing the websites of the 206 hospitals and eliminating 153 hospitals due to their lack of organizational chart
3. Accessing the organizational charts of the remaining 53 hospitals
4. Reviewing the last update of the organizational chart (2014–2021) of the 53 hospitals and eliminating 30 hospitals due to being out of date
5. Reviewing the remaining 23 charts and eliminating 15 hospitals due to the lack of efficient and effective organizational chart features such as organic, flexible, and flat charts, specialization, shorter hierarchies, and division of the specialized units [20]. Thus the organizational charts of 8 hospitals were systematically reviewed in the first phase of the study based on the stages mentioned above (S3 Fig)

Table 1 demonstrates the results of analyzing the organizational charts of hospitals from various countries. This table indicates that 8 hospitals from the Philippines, Canada, Egypt, the USA, and Iran entered the study and were investigated. The studied hospitals included general, single-specialty, and specialized hospitals in terms of the type of activity and teaching and non-teaching hospitals in terms of the type of task. The table includes the hospitals’ name, country, last update of the chart (year), task, activity, and complexity dimension based on the two criteria of vertical and horizontal separation. Hospitals were studied in terms of specialization and internal divisions of the organization in horizontal separation. On the other hand, the criteria of organizational hierarchy, supervision scope, number of organizational levels, and the shape of an organizational pyramid (flat, moderate, and tall) [20] were studied in vertical separation.

**Table 1:** Analysis of the organizational chart of selected hospitals.

No.	Hospital name	Chart update (year)	Country	Type of task and activity	Complexity	
					Horizontal separation criteria	Vertical separation criteria
1	Brockville General Hospital [24]	2021	Canada	General Non-teaching	**Specialization and internal organizational divisions:** In terms of the distinction and specialization of the duties, all the specialized units are under the respective deputy. Clinical units are under nursing service management, health information duties are under the information deputy, risk and project management are under the strategy and performance deputy, financial services are under the Deputy of Finance, and clinical department heads are under the supervisor of the board chairman.	**Organizational hierarchy:** *Top Managers (top of the pyramid*): Board of Directors *Senior Managers*: Executive Director *Middle managers*: 6 deputies, including Deputies of Nursing, Finance, Services, Health Information, Strategy and Performance, and Capital Project. The Head of Human Resources works directly under the Board of Directors. *Operational Managers*: Heads of units who are subordinate to middle managers. Thus: **The number of organizational levels**: The operational manager is two levels, and the chairman of the board is three levels away from the execution level. **Supervision scope**: Relatively wide **Organizational pyramid shape**: Moderate
2	San Lorenzo Ruiz general hospital [25]	2021	Philippines	Single-specialty Non-teaching	**Specialization and internal organizational divisions:** In terms of task distinction, all medical services are under one manager, specialized nursing services are under the nursing service manager, and all executive services, including financial and budgeting, human and support, and engineering medical equipment, are under the executive director’s supervisor.	**Organizational hierarchy:** *Top managers (top of the pyramid)*:H of the hospital *Senior Managers*: The nursing services manager, medical services manager, and executive director are under the head of the hospital. *Middle managers*: None *Operational Managers*: Heads of the units **The number of organizational levels**: The hierarchy is not long, and the head of the hospital is two management levels away from the operational level. **Supervision scope**: Wide since units are supervised by managers depending on their duties **Organizational pyramid shape**: Flat
3	Amin hospital [26]	2021	Iran	General teaching	**Specialization and internal organizational divisions:** Specialized units have not been charted properly, and nursing service management has been placed under the hospital manager’s subdivision rather than being assigned separate management under the hospital’s director despite its specialized tasks. However, all the specialized units work under the supervision of their respective deputy.	**Organizational hierarchy:** *Top management (top of the pyramid)*: The hospital director is at the top *Senior managers*: Deputy of education and research, hospital manager, and deputy of treatment working under the hospital director *Middle managers*: Nursing services, human resources, financial affairs, and support managers working under the hospital manager *Operational managers*: The heads of the units **The number of organizational levels**: The hospital director is three levels away from the operational managers working under the supervision of the hospital manager, and there is a distance of two levels between the hospital director and operational managers working under the deputies of research and treatment (in which case there are no middle managers) **Supervision scope**: Relatively wide **Organizational pyramid shape**: The chart is relatively moderate.
4	Shahid Dr. Chamran Heart Hospital [27]	2021	Iran	Single-specialty teaching	**Specialization and internal organizational divisions:** The study of this chart indicates a lack of proper charts in the units since nursing service management has been placed under the hospital manager’s subdivision rather than being assigned separate management despite its specialized tasks. Moreover, Paraclinical units are also under the supervision of the hospital manager despite their specialized activity.	**Organizational hierarchy**: *Top management (top of the pyramid)*: The hospital director is at the top *Senior managers*: deputy of education and research, hospital manager, and deputy of treatment working under the hospital director *Middle managers*: nursing manager and public affairs director (supervised by the hospital manager) *Operational Managers*: heads of units **The number of organizational levels**: The hospital director is three levels away from the operational managers working under the supervision of the hospital manager, and there is a distance of two levels between the hospital director and operational managers working under the public affairs director **Supervision scope**: Relatively wide **Organizational pyramid shape**: Moderate
5	Ganzouri specialized hospital [28]	2020	Egypt	Specialized Non-teaching	**Specialization and internal organizational divisions:** In terms of the distribution of duties, financial, support, human, legal, and information units are under the General Manager, while clinical, nursing, diagnostic, and treatment units that are among the specialized units of the hospitals are also under the General Manager despite requiring separate management.	**Organizational hierarchy:** *Top management (top of the pyramid)*: The executive director is on the top, and offices such as patient safety, nursing consultations, quality improvement, and medical consumables production consultation are under the executive director. *Senior managers*: The general manager who works under the executive director *Middle managers*: The heads of clinical wards (medical department), marketing manager, financial manager, legal affairs manager, information technology managers, human resource manager, and support service manager *Operational managers*: The heads of the units **The number of organizational levels**: The operational manager is three levels away from the top manager. **Supervision scope**: The supervision scope is relatively wide in this hospital. **Organizational pyramid shape**: Moderate
6	Shohada Yaft Abad hospital [29]	2019	Iran	General Non-teaching	**Specialization and internal organizational divisions:** The Paraclinical units are considered specialized in terms of activities but are placed under the executive deputy, so the duties have not been distributed properly in this sense. Some units such as hospital committees, patient safety, quality improvement, security, public relations, and risk and disaster management are directly under the supervision of the CEO, which can be delegated to senior managers.	**Organizational hierarchy:** *Top managers (top of the pyramid)*: The ECO is at the top of the pyramid *Senior Managers*: Technical and executive deputies and the nursing director are the senior managers working under the CEO’s supervision *Middle managers*: Heads of Paraclinical units and financial and administrative affairs working under the executive deputy, and deputy of treatment and physicians’ affairs director working under the technical deputy *Operational Managers*: Heads of units **The number of organizational levels**: The CEO is two management levels away from the operational level. **Supervision scope**: Wide **Organizational pyramid shape**: Flat
7	Strong Memorial Hospital [30]	2015	USA	Specialized teaching	**Specialization and internal organizational divisions:** Duties have been well distributed in these hospitals based on their specialization, and all nursing services are under the nursing director. Besides, all the affairs of physicians are under the supervision of the head of the medical department. All payment and financial activities are also under the respective manager’s supervision.	**Organizational hierarchy:** *Top management (top of the pyramid)*: The executive director is at the top *Senior managers*: Heads of nursing services, financial department, executive operations department, and medical department working under the executive director’s supervision *Middle managers*: none *Operational managers*: The heads of the units **The number of organizational levels**: The operational level is two levels away from the top manager. **Supervision scope**: Wide **Organizational pyramid shape**: Flat
8	Alexandra Marine & General Hospital [31]	2014	USA	General Non-teaching	**Specialization and internal organizational divisions:** Clinical wards work under the nursing executive director. Although the board of directors has a staff of consultants and the managing director has executive consultants as well, the physicians have no separate manager, and the support, diagnostic, and treatment units work under the chief operating manager despite the specialization of their jobs. It appears that diagnostic and treatment units need separate management, given their specialization.	**Organizational hierarchy:** *Top managers* ***(top of the pyramid)*:** The board of directors *Senior Managers*: CEO *Middle managers*: Chief operating manager and executive nursing manager *Operational Managers*: heads of clinical wards working under the nursing manager and heads of human resource, support, financial, information technology, and diagnostic and treatment units as well as the pharmacy working under the chief operating manager **The number of organizational levels**: The hierarchy is not long, and the head of the hospital is two management levels away from the operational level. **Supervision scope**: Wide **Organizational pyramid shape**: Flat

### 2-FGD

We examined organizational charts from Iran and various countries, and the initial organizational chart was designed using successful models in different countries such as America, Canada, Egypt, Philippines, and Iran, and organizational charts were designed for three types of hospitals. Based on the discussions in the FGD, is that organizations with flatter and more decentralized organizational charts tend to have faster decision-making, increased creativity, and higher levels of innovation. However, because measuring innovation and creativity is a complex task and the literature offers little evidence on how to measure an organization’s approach to innovation [23], therefore, we only analyzed the scope of supervision, the height of the organizational pyramid, and the complexity of horizontal and vertical separation [11].

In the designed chart, the hospital director is placed on top of the organization and supervises three to four deputies based on the nature of the hospital, while all the units fall under their respective deputy/manager based on the specialized tasks they need to report to them. The charts have been designed to take advantage of vertical and horizontal separation criteria seeking to decentralize, expand the supervision scope, and reduce bureaucracy, leading to increased decision-making speed and innovation in hospitals. S2 Fig illustrates an organizational chart of teaching, non-teaching, general, and single-specialty hospitals in Iran. As the diagram indicates, the organizational chart designed for Iranian hospitals has the following features:

1. The hospital director is placed at the top of the chart as the top manager in all these hospitals
2. The three deputies of research and education, treatment, and support have been considered the senior managers in teaching hospitals. The nursing services director goes alongside these three as a senior manager as well.
3. There is no deputy of research and education in non-teaching hospitals. In these hospitals, there is a director of treatment instead of the deputy of treatment and an executive director instead of the deputy of support, both of whom are considered senior managers.
4. Managers supervised by the deputy of support (in teaching hospitals) and executive director (in non-teaching hospitals) are considered middle managers.
5. Other units are the same in teaching and non-teaching hospitals, and unit managers are considered operational managers.
6. Single-specialty hospitals have clinical and treatment-diagnostic units that are absent from general hospitals. Financial, support, clinical, and treatment units are common between general and single-specialty hospitals.

Besides, it is observed in the complexity dimension based on the vertical and horizontal separation criteria that in terms of vertical separation and the height of the organizational pyramid, the units working under the deputies of treatment and research and education have two management levels with the unit manager as the operational manager, the deputies of treatment or research and education as the middle manager, and the hospital director as the top manager. However, units working under the deputies of support except for nutrition, the human resource department, the support department, the financial affairs department, the health information technology department, and the pharmacy have two management levels, while the aforementioned units have three management levels which are due to their greater diversity and specialization in terms of horizontal separation. In the case of nursing service management, the heads of outpatient and clinical nursing have three management levels (operational manager: head nurse of the clinical wards, middle manager: nursing affairs, senior manager: nursing service director, and top manager: head of the hospital) for the same reason, while other units working under this management have two management levels. In terms of horizontal separation, the units were divided and organized under various managers based on the iteration of tasks, the level of specialization, and the number of units. In this regard, administrative and support units were placed under the executive director/ deputy of support, nursing service units were placed under nursing management, treatment units were placed under the treatment manager/director, and research and education units were placed under the deputy of research and education. All units and offices were placed under the supervision of the respective manager or deputy based on the horizontal separation criteria.

## Discussion

A review of the organizational charts of a selection of Iranian hospitals indicated that in the case of Yaftabad Shohada Hospital, The Paraclinical units are considered specialized in terms of activities but are placed under the executive deputy. Hence, the duties have not been distributed properly in terms of horizontal separation and the criteria of specialization and internal organizational division. Specialized units have not been charted adequately in Amin Hospital either, and nursing service management has been placed under the hospital manager’s subdivision rather than being assigned separate management under the hospital’s director despite its specialized tasks. In Shahid Dr. Chamran Heart Hospital, the hospital director is three levels away from the operational managers working under the supervision of the hospital manager, and there is a distance of two levels between the hospital director and operational managers working under the public affairs director. Additionally, the principle of reducing organizational hierarchies has been complied with, but the principle of specialization has not, similar to the other two hospitals.

In the horizontal separation dimension, all the specialized units in Brockville General Hospital (Canada) are under the respective deputy given the two principles of specialization and internal organizational division, and the executive director at the senior manager level has six deputies, including Deputies of Nursing, Finance, Services, Health Information, Strategy and Performance, and Capital Project. Besides, all the units working under these deputies are connected in terms of task and specialty.

In San Lorenzo Ruiz General Hospital (Philippines), in terms of the distribution of duties, all medical services are under one manager, specialized nursing services are under the nursing service manager, and all executive services, including financial and budgeting, human and support, and medical equipment engineering are supervised by the executive director.

In Alexandra Marine & General Hospital (USA), clinical wards are also supervised by the nursing executive director. Clinical ward managers work under the nursing director, while the managers of human resource, support, financial, information technology, diagnostic-treatment, and pharmacy units are under the supervision of the senior operational manager.

Considering the above-mentioned notes, three deputies and one manager (senior managers), including the deputies of research and education, treatment, and support, and the nursing services manager, were designated and placed under the hospital director (top manager) in teaching hospitals. On the other hand, three managers (senior managers), including the treatment manager, nursing manager, and executive manager, were designated in non-teaching hospitals, while each unit was assigned to the respective manager or deputy based on their specialty and duties.

In the vertical separation dimension, considering the two principles of reducing the number of organizational hierarchy levels and increasing the supervision scope, the distance between the hospital director and operational level is two management levels in San Lorenzo Ruiz General Hospital (Philippines), while the top manager is three management levels away from the operational manager in Ganzouri specialized hospital (Egypt). Moreover, the executive director is two management levels away from the operational level in Strong Memorial Hospital (USA). The scope of supervision in the studied hospitals was wide or relatively wide, while their chart was flat or moderate, and in this study, the organizational chart for Iranian hospitals was designed given the advantages of the flat chart, including a wide scope of supervision, shorter hierarchy, specialization, and division of specialized units [20].

Various studies have been conducted on the structure and design of organizational charts. For instance, results of a 2022 qualitative study indicated that structural challenges included formality, complexity, centrality, environment, culture, and resources, and concluded that the appropriate structure needs to be designed and implemented on various levels considering their goals, current conditions, the challenges in the current organizational structure and the change in the goals and strategies of the Iranian healthcare system. Strategies and independence of management at hospitals and healthcare centers in towns can prepare these units to tackle the challenges in healthcare service provision [4]. In terms of decentralization, decision-making at top organizational levels and the need to design an appropriate organizational chart matching the challenges and goals of hospitals are consistent with the present study.

According to Michailidou (2021), the characteristics of fewer hierarchical levels, decentralization, involvement of executive managers, fewer organizational units, and horizontal coordination of organizational units can improve coordination in modern organizations [32]. These features are consistent with the organizational chart design criteria in the present study.

Another study by Torani et al. indicated that the goals left the greatest impact on the structural dimension [2]. This study was consistent with the present study in terms of its examination of the structural dimension and the influence of goals on it.

Results of another study suggested that managers pay more attention to centralization and delegation of authority among the other components of the structural dimension. Besides, most studies emphasize the need to modify the current organizational structure based on the macro goals and strategies of the healthcare system, which is consistent with the goals of the present study. The overview performed in the present study indicated that all the studies carried out in Iran have pointed to the satisfactory results of organizational structure decentralization [33], which is consistent with the present study’s recommendation to decentralize the units in the hospital chart.

## Conclusion

Based on a review of hospital organizational charts and FGD, the organizational chart for Iranian hospitals has been designed to have two management levels in terms of horizontal separation, the number of organizational levels, and organizational hierarchy, and to incorporate a wide scope of supervision and flat organizational pyramid shape, reducing the distance between the managers and employees and removing the redundant management levels. In terms of the horizontal separation dimension, including the two principles of specialization and internal organizational division, the units were organized and divided based on the iteration of duties, the level of specialization, and the number of units, and each specialized unit was placed under the respective manager. Using the same organizational chart for hospitals by considering whether they are teaching, non-teaching, general, or single-specialty hospitals can lead to procedural unity in the implementation of the Ministry of Health’s decisions by the units in hospitals and fairer accreditation scores for the hospitals.

The present investigation’s outcomes give supervisors and policymakers the opportunity to revamp the organizational chart of Iranian hospitals by their obligations and actions. This, in turn, would promote consistency in the nomenclature of the hospitals based on the guidance provided in the "Collection of Organizational Engineering Regulations and Engineering and Job Evaluation" that has been released by the Iranian Ministry of Health and Medical Education. Subsequent research endeavors are recommended to scrutinize the content facet in the development of hospital organizational charts across different nations. Furthermore, it is suggested to formulate hospital hierarchical frameworks while taking into account the aspects of formality and centralization.

### Limitation

Face to face FGD with experts who were located in different parts of the country can take up a lot of time and money. To overcome this limitation, virtual methods were used to conduct FGD.

## Supporting information

S1 Checklist COREQ (Consolidated criteria for REporting Qualitative research) checklist. (DOCX)

S1 Fig Study design. (DOCX)

S2 Fig Organizational chart of teaching, non-teaching, general, and single-specialty hospitals in Iran. (DOCX)

S3 Fig The process of hospital organizational charts selection for the overview. (DOCX)

## Abbreviation

FGD: Focus Group Discussion
