# Supplementary material for: Designing Iranian hospital organizational charts: Global comparisons
Source: PLoS One. 2024 Mar 27;19(3):e0300985. doi: 10.1371/journal.pone.0300985 (PMC10971672; doi:10.1371/journal.pone.0300985)
Supplement: S1 Checklist — (DOCX) [file pone.0300985.s001.docx]

**COREQ (Consolidated criteria for REporting Qualitative research) Checklist**

A checklist of items that should be included in reports of qualitative research. You must report the page number in your manuscript

where you consider each of the items listed in this checklist. If you have not included this information, either revise your manuscript

accordingly before submitting or note N/A.

**Topic**

**Item No.**

**Guide Questions/Description**

**Reported on**

**Page No.**

**Domain 1: Research team**

**and reﬂexivity**

*Personal Characteristics*

Interviewer/facilitator

Credentials

Occupation

Gender

Experience and training

*Relationship with*

*participants*

1

2

3

4

5

Which author/s conducted the interview or focus group? 6

What were the researcher’s credentials? E.g. PhD, MD 6

What was their occupation at the time of the study? 6

Was the researcher male or female? 6

What experience or training did the researcher have? 6

Relationship established

Participant knowledge of

the interviewer

6

7

Was a relationship established prior to study commencement? 6

What did the participants know about the researcher? e.g. personal 6

goals, reasons for doing the research

Interviewer characteristics

8

What characteristics were reported about the interviewer/facilitator? 6

e.g. Bias, assumptions, reasons, and interests in the research topic

**Domain 2: Study design**

*Theoretical framework*

Methodological orientation

and Theory

9

What methodological orientation was stated to underpin the study? e.g. 6

grounded theory, discourse analysis, ethnography, phenomenology,

content analysis

*Participant selection*

Sampling

10

11

How were participants selected? e.g. purposive, convenience, 6

consecutive, snowball

How were participants approached? e.g. face-to-face, telephone, mail, 6

email

Method of approach

Sample size

Non-participation

*Setting*

12

13

How many participants were in the study? 6

How many people refused to participate or dropped out? Reasons? 6

Setting of data collection

Presence of non-

participants

14

15

Where was the data collected? e.g. home, clinic, workplace  **N**/A

Was anyone else present besides the participants and researchers?  **N**/A

Description of sample

16

17

What are the important characteristics of the sample? e.g. demographic 6

data, date

*Data collection*

Interview guide

Were questions, prompts, guides provided by the authors? Was it pilot 6

tested?

Repeat interviews

Audio/visual recording

Field notes

Duration

Data saturation

Transcripts returned

18

19

20

21

22

23

Were repeat interviews carried out? If yes, how many? 6

Did the research use audio or visual recording to collect the data? 6

Were ﬁeld notes made during and/or after the inter view or focus group? 6

What was the duration of the inter views or focus group? 6

Was data saturation discussed? 6

Were transcripts returned to participants for comment and/or 6

**Topic**

**Item No.**

**Guide Questions/Description**

**Reported on**

**Page No.**

correction?

**Domain 3: analysis and**

**ﬁndings**

*Data analysis*

Number of data coders

Description of the coding

tree

24

25

How many data coders coded the data? **N**/A

Did authors provide a description of the coding tree? **N**/A

Derivation of themes

Software

Participant checking

*Reporting*

26

27

28

Were themes identiﬁed in advance or derived from the data? **N**/A

What software, if applicable, was used to manage the data? **N**/A

Did participants provide feedback on the ﬁndings? 6

Quotations presented

29

Were participant quotations presented to illustrate the themes/ﬁndings? **N**/A

Was each quotation identiﬁed? e.g. participant number

Data and ﬁndings consistent

Clarity of major themes

Clarity of minor themes

30

31

32

Was there consistency between the data presented and the ﬁndings? **N**/A

Were major themes clearly presented in the ﬁndings? **N**/A

Is there a description of diverse cases or discussion of minor themes? **N**/A

Developed from: Tong A, Sainsbury P, Craig J. Consolidated criteria for reporting qualitative research (COREQ): a 32-item checklist

for interviews and focus groups. *International Journal for Quality in Health Care*. 2007. Volume 19, Number 6: pp. 349 – 357

s
