# Supplementary material for: Designing Iranian hospital organizational charts: Global comparisons
Source: PLoS One. 2024 Mar 27;19(3):e0300985. doi: 10.1371/journal.pone.0300985 (PMC10971672; doi:10.1371/journal.pone.0300985)
Supplement: S1 Fig — (DOCX) [file pone.0300985.s002.docx]

**Diagram1: Study design**

***Step1:* *Data collection***

1. Searching hospital websites
2. Examination of the organizational chart available on hospital websites
3. Checking whether the organizational chart is up-to-date
4. Examining the organizational chart based on the specifications of effective and efficient charts

**Phase one**

***Step 2*: *Data analysis***

All the selected organizational charts were analyzed, and the initial hospital organization chart was designed

***Step1:* *FGD design***

1. The main goal behind the design of an organizational chart was specified
2. A list of the features of appropriate and efficient organizational charts was prepared as a guide for each session of the FGD.
3. 8 experts were selected based on inclusion criteria
4. The results of the first phase (overview) and the basis for the design of hospital organizational charts were explained to the experts, and each was asked to express their individual opinions

**Phase two**

***Step2: Data collection***

Experts' opinions regarding the organizational position of each unit based on the vertical and horizontal separation criteria were shared over 12 two-hour sessions.

***Step3:* *Analysis and Conclusion***

1. The initial chart designed in the previous stage was demonstrated and explained to focus group discussion members to vote and rank their opinions.
2. The members discussed the initial chart over three two-hour sessions. Thus, the final chart was compiled.
3. Consensus was reached on opposing opinions through voting, and dominant opinions were determined based on voting results.

***Step4: Report compilation***

Results of the FGD sessions were integrated and compiled in a report. All the opinions were shared in a process called member review to validate the results, which increased the validity of the designed chart. Also, COREQ checklist was used to ensure a clear and comprehensive report
