# Supplementary material for: Designing Iranian hospital organizational charts: Global comparisons
Source: PLoS One. 2024 Mar 27;19(3):e0300985. doi: 10.1371/journal.pone.0300985 (PMC10971672; doi:10.1371/journal.pone.0300985)
Supplement: S2 Fig — (DOCX) [file pone.0300985.s003.docx]

**Diagram 2: Organizational chart of teaching, non-teaching, general, and single-specialty hospitals in Iran**


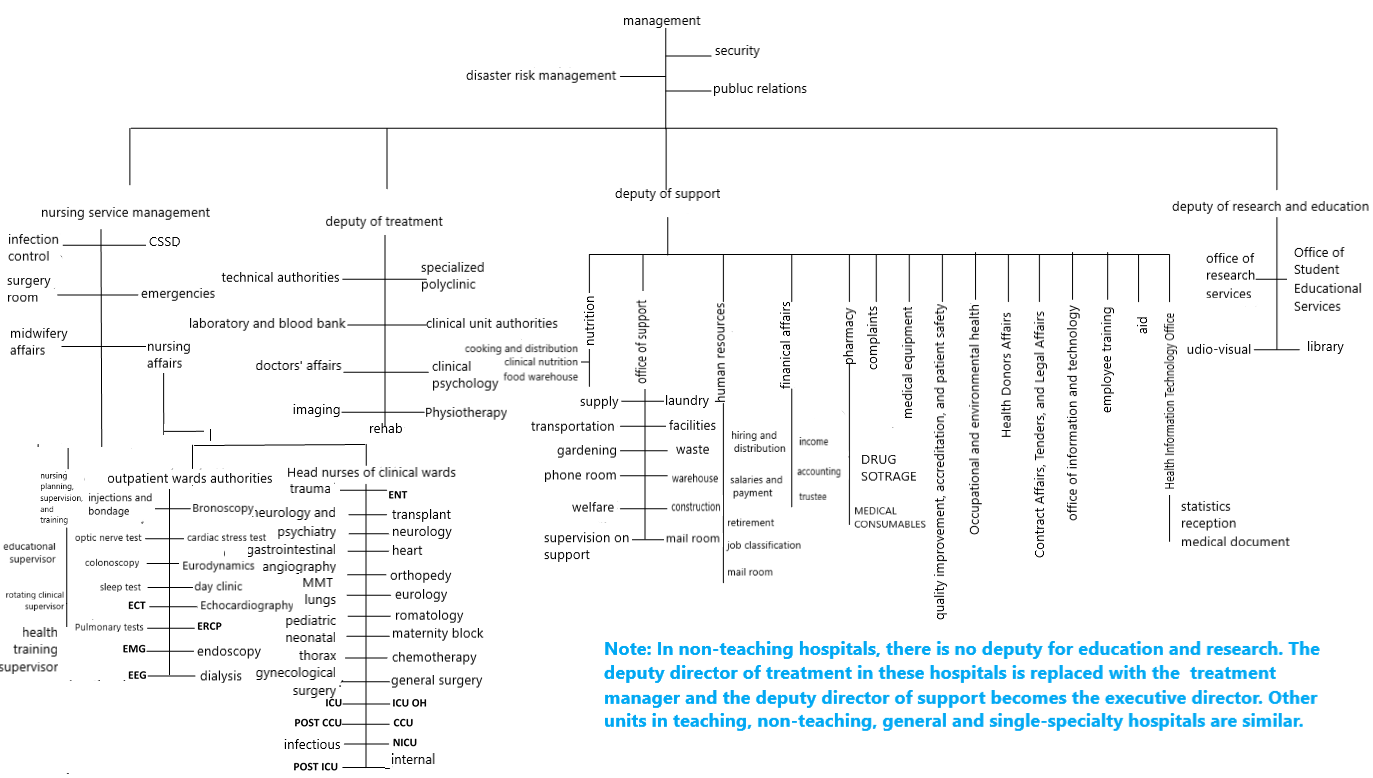


ERCP: Endoscopic Retrograde Cholangiography ENT: Ear Nose and Throat ICU: Intensive Care Unit CCU: Coronary Care Unit ECT: Electro Convulsive Theraphy EMG: Electromyography EEG: Electroencephalography CSSD: Central Sterilization Supply Department ICU OH: Intensive Care Unit Open Heart Post ICU: Post Intensive Care Unit Post CCU: Post Coronary Care Unit NICU: Neonatal Intensive Care Unit
