# Supplementary material for: Designing Iranian hospital organizational charts: Global comparisons
Source: PLoS One. 2024 Mar 27;19(3):e0300985. doi: 10.1371/journal.pone.0300985 (PMC10971672; doi:10.1371/journal.pone.0300985)
Supplement: S3 Fig — (DOCX) [file pone.0300985.s004.docx]

Accessing the organizational charts of hospitals (n=53)

Accessing the websites of hospitals (n=206)

Elimination of hospitals due to their lack of organizational chart (n=153)

Elimination of hospitals due to being out of date (n=30)

Reviewing the last update of the organizational chart (2014-2021) of the hospitals (n=23)

Elimination of hospitals due to the lack of efficient and effective organizational chart features such as organic, flexible, and flat chart, specialization, shorter hierarchies, and division of the specialized units (n=15)

Including the selected organizational charts into the overview (n=8)

**Figure1. The process of hospital organizational charts selection for the overview**
